# Genome-wide identification of the BES1 gene family and functional study of *VcBES1.3* under salt stress in blueberry (*Vaccinium corymbosum*)

**DOI:** 10.3389/fpls.2026.1965422

**Published:** 2026-10-13

**Authors:** Baofeng Zhang, Xueying Liu, Jiahuan Huang, Hong Su, Xuan Luo, Ye Sun, Fengjun Zhao, Xiaochen Lu, Shan Lin, Donglai Wang, Guohui Huang, Wenjie Zhou

**Affiliations:** College of Agriculture, Liaodong University, Dandong, Liaoning, China

**Keywords:** BES1, bioinformatics analysis, salt, Vaccinium corymbosum, VcBES1.3

## Abstract

BES1 (BRI1-EMS-SUPPRESSOR 1) transcription factors mediate brassinosteroid signaling and regulate plant growth, development, and stress responses. The BES1 family has not been characterized in blueberry (*Vaccinium corymbosum* L.). We identified 16 *VcBES1* family members and classified them into four groups based on phylogenetic analysis. Promoters of the BES1 genes mainly contain *cis*-elements associated with abiotic stress and phytohormone signaling. Tissue-specific expression analysis showed that the *VcBES1* family is expressed predominantly in roots and stems. All members except *VcBES1.8* and *VcBES1.9* responded positively to salt stress. Among them, *VcBES1.3* displayed the strongest salt induction. The protein accumulated in the nucleus. Overexpression and RNA interference (RNAi) experiments established that *VcBES1.3* enhances salt tolerance. This study characterizes the *VcBES1* family, defines its tissue-specific and stress-responsive expression patterns, and demonstrate that *VcBES1.3* as a positive regulator of salt tolerance in blueberry.

## 1 Introduction

Blueberry (*Vaccinium corymbosum* L, Ericaceae) accumulates abundant anthocyanins and other polyphenolic metabolites, bioactive compounds widely documented to confer multiple human health benefits (Prior et al., 1998; Kalt et al., 2020). As a shallow-rooted crop, blueberry exhibits stringent requirements for soil acidity, water supply and mineral nutrition. It can only develop normally under acidic substrates with pH values ranging from 4.5 to 5.5, and is highly susceptible to salt stress. With the rapid expansion of protected blueberry production, continuous salt accumulation in root zones under closed fertigation systems has become a major constraint limiting fruit yield and quality (Munns and Tester, 2008). Salt stress imposes two successive adverse effects on blueberry plants. At the early stage, osmotic stress occurs and induces cellular dehydration. Long-term salt exposure further causes Na^+^ cytotoxicity and excessive accumulation of reactive oxygen species (ROS), which disrupt cell membrane integrity and damage photosynthetic structures, eventually inducing programmed cell death and suppressing plant vegetative growth (Fu and Yang, 2023). Therefore, isolating and functionally characterizing key salt-tolerant genes in blueberry is critical for germplasm improvement and sustainable industrial cultivation.

Brassinosteroids (BRs) are plant-specific sterol hormones. They represent the sixth major class of plant hormones, following the earlier discoveries of auxin, gibberellin, cytokinin, abscisic acid, and ethylene. BRs govern germination, cell elongation, vascular patterning, photomorphogenesis, reproductive development, senescence, and stress responses (Clouse and Sasse, 1998; Peres et al., 2019; Nolan et al., 2020). Disruption of BR homeostasis produces dwarf plants with reduced fertility and compromised stress tolerance (Kim and Wang, 2010; Nolan et al., 2020). BR signaling ultimately targets two transcription factors: BRI1-EMS-SUPPRESSOR 1 (BES1) and BRASSINAZOLE-RESISTANT 1 (BZR1). *BES1* and *BZR1* are regulated by a simple phosphorylation switch. Under BR-limiting conditions, upstream kinases phosphorylate both proteins, sequestering them in the cytoplasm and blocking their transcriptional activity. BR perception at *BRI1* triggers a dephosphorylation cascade that reactivates the two factors, allowing them to translocate into the nucleus. Once there, *BES1* and *BZR1* recognize *E*-boxes and BRREs in target gene promoters and regulate the expression of thousands of downstream genes (He et al., 2005; Yu et al., 2011; Nolan et al., 2020). *BZR1* and *BES1* do not act in isolation. They interface with ABA signaling through BIN2, a BR pathway kinase that directly phosphorylates the ABA kinase SnRK2, and contribute to stress memory through epigenetic mechanisms (Bulgakov and Avramenko, 2020). In crops such as soybean and tomato, BES1 family homologs mediate hormone responses and salt stress tolerance (Song et al., 2019; Jia et al., 2021), placing this transcription factor family at the center of the growth defense trade-off.

The BES1 family has been identified in a wide range of plant genomes, though the number of members differs sharply across species. Among monocots, 6 genes have been reported in rice (*Oryza sativa*) and 19 in wheat (*Triticum aestivum*) (Cheng et al., 2023; Zhang et al., 2025b). The dicot side of the family is considerably larger and more variable: 6 members in *Arabidopsis thaliana* and melon (*Cucumis melo*), 8 in grape (*Vitis vinifera*), 9 in tomato (*Solanum lycopersicum*), 10 in tea *(Camellia sinensis*), 16 in soybean (*Glycine max*), 22 in apple (*Malus domestica*), and up to 28 in Brassica napus (Song et al., 2018; Li et al., 2019, 2023; Cao et al., 2020; Su et al., 2021; Yang et al., 2023; Zhang et al., 2025c, 2025a). No BES1 sequences have been detected in cyanobacteria or red algae. A pan-genome survey covering 27 plant species recovered 135 BES1 sequences in total, all restricted to land plants, which places the origin of the family after the divergence of the algal lineage (Sun et al., 2022). It is therefore likely that BES1 emerged during the transition to terrestrial life and may have contributed to the evolution of stress adaptation on land. BES1 proteins are not limited to BR signal transduction. *OsBES1–3* and *OsBES1–5* promote root elongation under drought in rice (Sun et al., 2022). *SlBES1* directly targets carotenoid biosynthesis genes and helps drive fruit ripening in tomato (Su et al., 2021). *ZmBES1/BZR1-1*, by contrast, appears to suppress drought tolerance in maize (Feng et al., 2023b). A connection between BES1 and salt tolerance has also emerged. The *Arabidopsis bes1-D* gain-of-function mutant performs better than the wild type under salt stress. At least part of the mechanism is known: BES1 binds the promoters of NHX2 and CAX3 (encoding ion transporters) and upregulates both genes, helping to preserve ion homeostasis (Feng et al., 2023a). In wheat, salt treatment induces *TaBES1* expression, and the magnitude of induction correlates with cultivar-level differences in salt tolerance (Wang et al., 2023). Alfalfa tells much the same story: most BES1 family members are upregulated by salt and drought (Chen et al., 2024). Across monocot and dicot species, BES1 has now been repeatedly linked to salt adaptation, suggesting the connection is evolutionarily old rather than lineage-specific (Cao et al., 2024; Li et al., 2025). In blueberry, however, the BES1 family has not been systematically characterized, and whether any of its members contribute to salt tolerance is unknown. With a high-quality genome annotation now available (Yocca et al., 2023), identifying the full set of BES1 genes and testing their roles under salt stress is important.

We recovered 16 *VcBES1* genes from the tetraploid blueberry genome and classified them into four phylogenetic groups. Their gene structures, conserved motifs, promoter *cis*-elements, and syntenic relationships were characterized. Transcriptome data identified *VcBES1.3* as the family member with the greatest induction under both drought and salt stress, and the protein localizes to the nucleus. Overexpression of *VcBES1.3* enhanced salt tolerance. RNAi silencing of *VcBES1.3* suppressed antioxidant enzyme induction under salt stress; ROS accumulated and membrane lipids were peroxidized. Collectively, *VcBES1.3* is a positive regulator of salt tolerance in blueberry.

## 2 Materials and methods

### 2.1 Identification and physicochemical characterization of VcBES1 proteins

*Arabidopsis thaliana* BES1 protein sequences (*BES1*, *AT1G19350*; *BZR1*, *AT1G75080*; *BEH1*, *AT3G50750*; *BEH2*, *AT4G36780*; *BEH3*, *AT4G18890*; *BEH4*, *AT1G78700; BMY2, AT5G45300; BMY4, AT2G45880*) were retrieved from TAIR (https://www.arabidopsis.org/) and used as BLASTp queries against the *V. corymbosum* ‘Duke’ genome (Colle et al., 2019) with an *E*-value threshold of 1e-5. The Hidden Markov Model profile for the BES1_N domain (PF05687) was obtained from the Pfam database for HMMER search (Eddy, 2011). Candidate sequences were validated through NCBI Conserved Domain Search and SMART (Letunic et al., 2021). After removing redundant sequences, 16 non-redundant *VcBES1* genes were retained. Physicochemical properties were computed using the ExPASy ProtParam tool (https://web.expasy.org/protparam/).

### 2.2 Phylogenetic analysis

Full-length BES1 protein sequences from *V. corymbosum*, *A. thaliana*, *P. trichocarpa*, and *S. lycopersicum* were aligned using ClustalW software. A neighbour-joining (NJ) phylogenetic tree was constructed using MEGA 11 (Tamura et al., 2021) with the JTT matrix-based model and 1000 bootstrap replicates. The tree was visualised and annotated using iTOL (Letunic and Bork, 2021).

### 2.3 Conserved Motif, domain, and gene structure analysis

Conserved motifs were identified using MEME Suite v5.5.0 (Bailey et al., 2009) with a maximum of 10 motifs. Conserved domain architecture was validated using Pfam and NCBI Batch CD-Search. Gene structures were extracted from genome annotation files and visualised with TBtools v2.0 (Chen et al., 2023).

### 2.4 Promoter cis-regulatory element analysis

The 2, 000-bp sequences upstream of the translation start codon of each *VcBES1* gene were extracted from the blueberry genome and submitted to PlantCARE (Lescot, 2002). Identified *cis*-elements were classified into stress-responsive, hormone-responsive, and development-related categories based on functional annotations. The distribution of *cis*-elements was visualized using TBtools-II v2.0 (Chen et al., 2023).

### 2.5 Intragenomic and interspecies synteny analysis

Intragenomic collinearity among VcBES1 genes was analysed using MCScanX (Wang et al., 2012) with default parameters. Ka/Ks ratios were calculated for duplicated gene pairs using the Nei-Gojobori method implemented in TBtools. Interspecies collinearity was assessed between blueberry and tomato (*S. lycopersicum* SL3.0), *Arabidopsis* (*A. thaliana* TAIR10), and poplar (*P. trichocarpa* v4.0) using MCScanX. Collinearity blocks were visualised using TBtools. In addition, duplication modes were validated with DupGen_finder (Qiao et al., 2019), which classifies duplicated gene pairs into five modes: WGD, tandem, proximal, transposed, and dispersed.

### 2.6 Plant materials and stress treatments

Blueberry (*V. corymbosum* ‘Duke’) tissue-cultured plantlets were maintained on half-strength Murashige and Skoog (MS) medium at 25 °C under a 16 h light/8 h dark photoperiod. One-year-old potted seedlings of ‘Duke’ were grown in a walk-in growth chamber with controlled environmental conditions (21-25 °C, 16 hours of light/8 hours of dark, and 60-80% humidity). These potted seedlings were used for stress treatments and tissue expression profiling. For drought stress, watering was withheld for 5 days; control plants received normal irrigation. For salt stress, plants were irrigated with 200 mM NaCl solution for 5 days. Leaf samples from both stressed and control plants were collected at the same time, frozen immediately in liquid nitrogen, and stored at −80 °C. For each condition, three independent biological replicates were collected, each consisting of leaf tissue from a single plant.

Salt concentrations and treatment durations for each experiment were determined in preliminary trials. A severe regime (200 mM NaCl for 5 days) was used to induce a strong transcriptional response in wild-type plants for transcriptome analysis, whereas a milder regime (150 mM NaCl) was used for phenotypic assays because it caused only mild visible symptoms in wild-type plants, thereby providing a clear window for detecting differences in stress sensitivity among genotypes.

### 2.7 RNA extraction and transcriptome analysis

Total RNA was extracted using the RNAprep Pure Plant Kit (TIANGEN, Beijing, China). RNA-seq libraries were constructed and sequenced on the Illumina HiSeq™X-Ten platform (150 bp paired-end reads). Clean reads were mapped to the *V. corymbosum* reference genome using HISAT2 (Kim et al., 2019). Read counts per gene and transcript abundance (TPM) were quantified with StringTie (Pertea et al., 2015). DESeq2 (Love et al., 2014) was used to identify differentially expressed genes between the control and stress-treated groups (adjusted *P*-value < 0.05). For heatmap visualisation only, log_2_ fold changes between treatment and control groups were calculated from mean TPM values with a pseudocount of 0.01 added to handle zero values. Heatmaps were generated using TBtools.

### 2.8 Subcellular localisation

The coding sequence of *VcBES1.3* lacking the stop codon was cloned into the pCAMBIA1300-35S:GFP vector to generate a C-terminal GFP fusion. The construct was transformed into *Agrobacterium tumefaciens* strain GV3101 and infiltrated into leaves of 4-week-old *N. benthamiana* plants. The nuclear marker 35S:H2B-mCherry was co-infiltrated. Fluorescence was observed 48–72 h post-infiltration using a confocal laser scanning microscope (Nikon AX R, Tokyo, Japan).

### 2.9 Generation of transgenic *V. corymbosum*

The coding sequence of *VcBES1.3* was cloned into the pBI121 vector under the CaMV 35S promoter. Additionally, both the sense and antisense fragments of *VcBES1.3* were inserted into the pRI101 vector to construct the 35S:VcBES1.3-RNAi suppression vector. All vectors were transformed into *Agrobacterium tumefaciens* (GV3101) for plant transformation. Stable transgenic blueberry lines were generated by *Agrobacterium-mediated* leaf disc transformation as described by Song (Song, 2015). Putative transgenic seedlings were screened by genomic PCR, and transgene expression was verified by RT-qPCR. The primer sequences employed in this study are detailed in **Supplementary Table S1**.

### 2.10 Real-time quantitative PCR

cDNAs were synthesized from total RNA using the TaKaRa Reverse Transcription Kit (TaKaRa, RR014A). RT-qPCR was performed with FastStart Universal SYBR Green Master Mix (Roche, 4913914001) on an Agilent Mx3000P Real-Time PCR System. Both kits were used according to the manufacturer’s instructions. The gene expression was normalized to the expression of the *VcUBC28* gene. The primers used for RT-qPCR are listed in **Supplementary Table S1**.

### 2.11 Salt tolerance assay

Four independent overexpression lines and two RNAi lines were obtained. Line *OE-VcBES1.3-2#*, with the highest transcript level, and the RNAi lines (*RNAi-VcBES1.3-1#* and *RNAi-VcBES1.3-2#*) were used for phenotypic assays. *In vitro* plantlets (WT and *OE-VcBES1.3-2#*) were grown on half-strength MS medium containing 0, 10, 50, 100, or 150 mM NaCl. Fresh weight was recorded after 60 days. Biomass loss was calculated as: Biomass loss (%) = [(FW_control − FW_salt)/FW_control] × 100. Four-month-old WT and RNAi seedlings were irrigated with 150 mM NaCl for 12 days. Leaf samples were collected at day 0 and day 12 for physiological measurements.

### 2.12 Physiological and biochemical measurements

Leaf samples were collected after 12 days of salt treatment. Malondialdehyde (MDA) content was measured using the thiobarbituric acid method (Heath and Packer, 1968). Hydrogen peroxide (H_2_O_2_) content was determined following Velikova (Velikova et al., 2000). Superoxide dismutase (SOD) activity was assayed by the nitroblue tetrazolium reduction method (Giannopolitis and Ries, 1977). Peroxidase (POD) activity was measured using the guaiacol oxidation method. Catalase (CAT) activity was determined by monitoring the decomposition of H_2_O_2_ at 240 nm (Aebi, 1984). For each assay, three independent biological replicates were analysed per genotype per treatment; each biological replicate consisted of pooled leaf tissue from three plants, which was ground to a fine powder in liquid nitrogen and thoroughly mixed before analysis.

### 2.13 Statistical analysis

All experiments were performed with three independent biological replicates, defined for each assay in the corresponding Methods sections. Data are presented as means ± SEM. For the biomass assay (genotype × NaCl concentration), data were analysed by two-way ANOVA followed by Tukey’s HSD test. For the physiological and biochemical assays, data were analysed by one-way ANOVA within each treatment, and for the qRT-PCR specificity analysis by one-way ANOVA across genotypes, both followed by Tukey’s HSD test. Pairwise comparisons between two groups were assessed using two-tailed Student’s t-tests where applicable. All statistical analyses were performed using SPSS v26.0.

## 3 Results

### 3.1 Identification and characterization of the *VcBES1* gene family in blueberry

To identify BES1 family genes in blueberry, BLAST searches were performed against the *V. corymbosum* cv. *Duke, v1* genome database using ATBES1 protein sequences. Following Pfam analysis, HMMER prediction, and two rounds of conserved domain filtering, 16 BES1 family genes were finally identified and designated as *VcBES1.1* to *VcBES1.16* based on their order along the scaffolds (**Table 1**). Physicochemical property analysis of these 16 VcBES1 members showed that their amino acid lengths ranged from 108 to 328, with molecular weights spanning of 12.27–35.64 kDa. The theoretical isoelectric points (pI) ranged from 7.61 to 9.47, indicating a predominantly basic nature. All members had instability indices greater than 40 (some exceeding 84), indicative of intrinsically unstable proteins. The aliphatic indices ranged from 52.34 to 69.81, and the grand average of hydropathicity (GRAVY) values were all negative (from –1.087 to –0.521), demonstrating that all VcBES1 proteins are hydrophilic.

**Table 1:** Identification and physicochemical properties of the 16 BES1 transcription factor family members in blueberry.

Gene name	Locus name	Amino acid no.	Molecular weight (Da)	Theoretical pI	Instability index	Aliphatic index	GRAVY
*VcBES1.1*	Vc_DUK_00059260-RA	192	20495.81	9.44	47.21	63.07	-0.617
*VcBES1.2*	Vc_DUK_00055259-RA	158	17311.5	7.74	50.96	69.81	-0.551
*VcBES1.3*	Vc_DUK_00028162-RA	314	34028.04	9.04	71.04	54.14	-0.701
*VcBES1.4*	Vc_DUK_00008244-RA	151	17546.85	7.65	84.17	62.72	-1.02
*VcBES1.5*	Vc_DUK_00041070-RA	328	35643.91	8.9	71.74	56.89	-0.684
*VcBES1.6*	Vc_DUK_00024946-RA	192	20483.76	9.44	49.5	61.04	-0.641
*VcBES1.7*	Vc_DUK_00014732-RA	192	20467.76	9.44	50.59	62.08	-0.63
*VcBES1.8*	Vc_DUK_00032698-RA	198	23357.44	8.56	76.59	60.66	-1.087
*VcBES1.9*	Vc_DUK_00026211-RA	108	12268.8	9.47	43.24	54.17	-1.056
*VcBES1.10*	Vc_DUK_00035767-RA	298	32048.8	9.09	84.1	58.36	-0.524
*VcBES1.11*	Vc_DUK_00035777-RA	298	32061.8	9.09	84.1	58.36	-0.534
*VcBES1.12*	Vc_DUK_00063313-RA	298	32014.78	9.09	84.61	59.66	-0.521
*VcBES1.13*	Vc_DUK_00037814-RA	325	34705.61	8.06	56.42	53.23	-0.591
*VcBES1.14*	Vc_DUK_00088554-RA	151	17546.85	7.65	84.17	62.72	-1.02
*VcBES1.15*	Vc_DUK_00067935-RA	314	34028.04	9.04	71.04	54.14	-0.701
*VcBES1.16*	Vc_DUK_00048756-RA	325	34678.16	7.61	56.3	52.34	-0.607

To explore the phylogenetic relationship among the VcBES1 proteins, we analyzed a total of 47 BES1 proteins, which were derived from *Arabidopsis* (8), *poplar* (14), and *tomato* (9). VcBES1 proteins were divided into four groups and exhibited close relatedness to heterologous BES1 homologs, consistent with the high conservation of this family throughout plant evolution (**Figure 1**). Group 4 was the largest, with seven members clustering near the canonical *Arabidopsis* BES1 (*AT1G19350*) and BZR1 (*AT1G75080*). Groups 1 and 2 contained three and four members, respectively. The two Group 3 members associated with AtBEH3 (*AT4G18890*) and AtBEH4 (*AT1G78700*). Each of the four *Arabidopsis* BES1 subfamilies received at least one blueberry representative.

**Figure 1:**
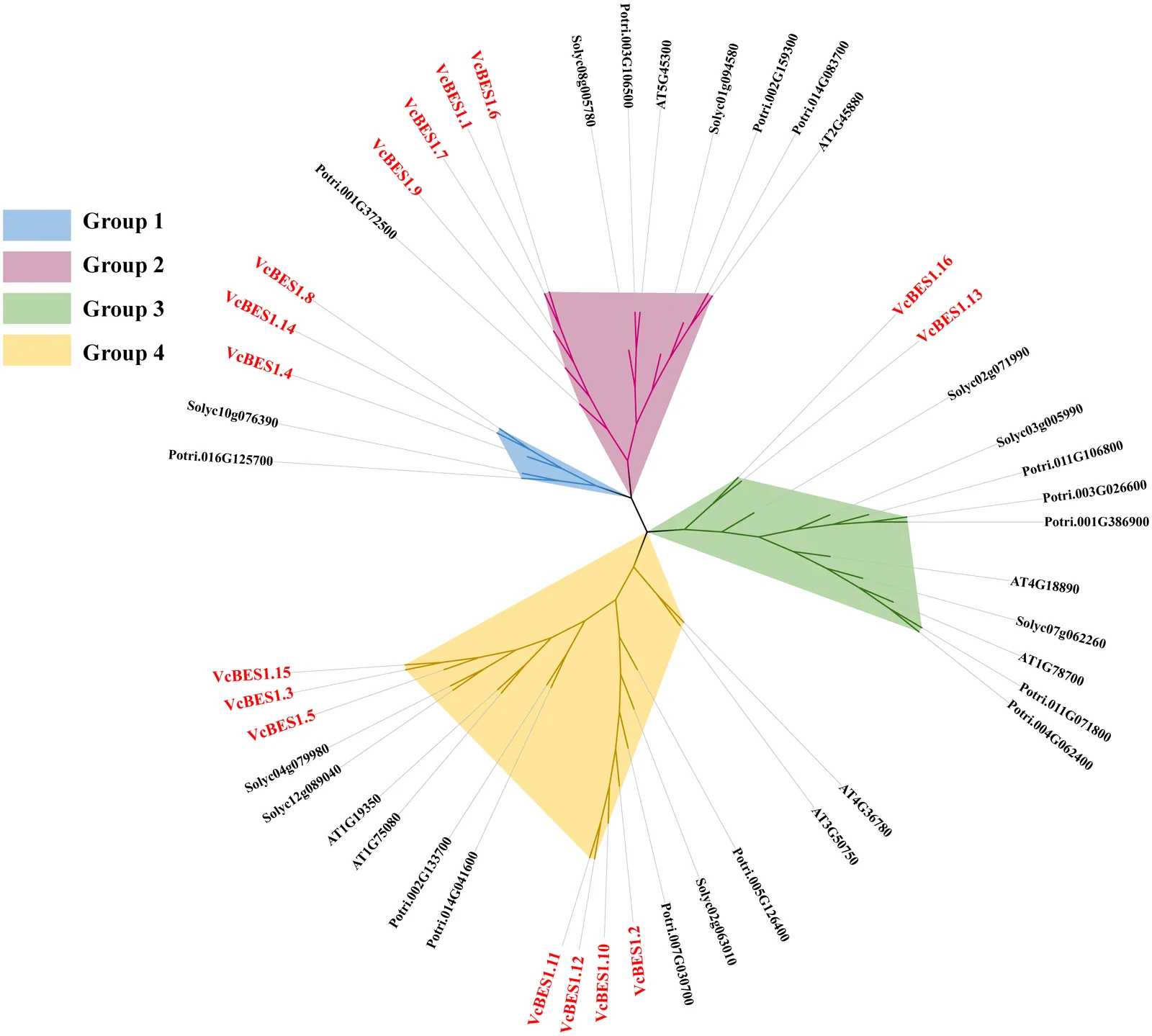
Phylogenetic tree of BES1 family proteins derived from *Vaccinium corymbosum*, *Solanum lycopersicum*, *Arabidopsis thaliana*, and *Populus trichocarpa*. Blue polygon indicates Group 1; purple polygon indicates Group 2; green polygon indicates Group 3; yellow polygon indicates Group 4.

### 3.2 Conserved motifs and gene structure analysis of the VcBES1 family

To further characterize the *VcBES1s*, we analyzed their conserved motifs, functional domains, and gene structures in combination with a phylogenetic tree based on full-length VcBES1 protein sequences (**Figure 2A**). MEME analysis identified a total of 10 conserved motifs, designated Motifs 1 to 10. Motif 1 corresponded to the DNA-binding region and occupied the N-terminus of every member, representing the BES1_N superfamily domain (**Figures 2B, C**). Motifs 3, 6, 7, and 10 were located immediately downstream of Motif 1 in a largely fixed order, forming a compact N-terminal module (**Figure 2B**). Analysis of gene structures revealed that nearly all VcBES1 genes consisted of two exons split by a single intron, except *VcBES1.5* with three exons (**Figure 2D**). This minimalist gene architecture is conserved throughout angiosperm BES1 families.

**Figure 2:**
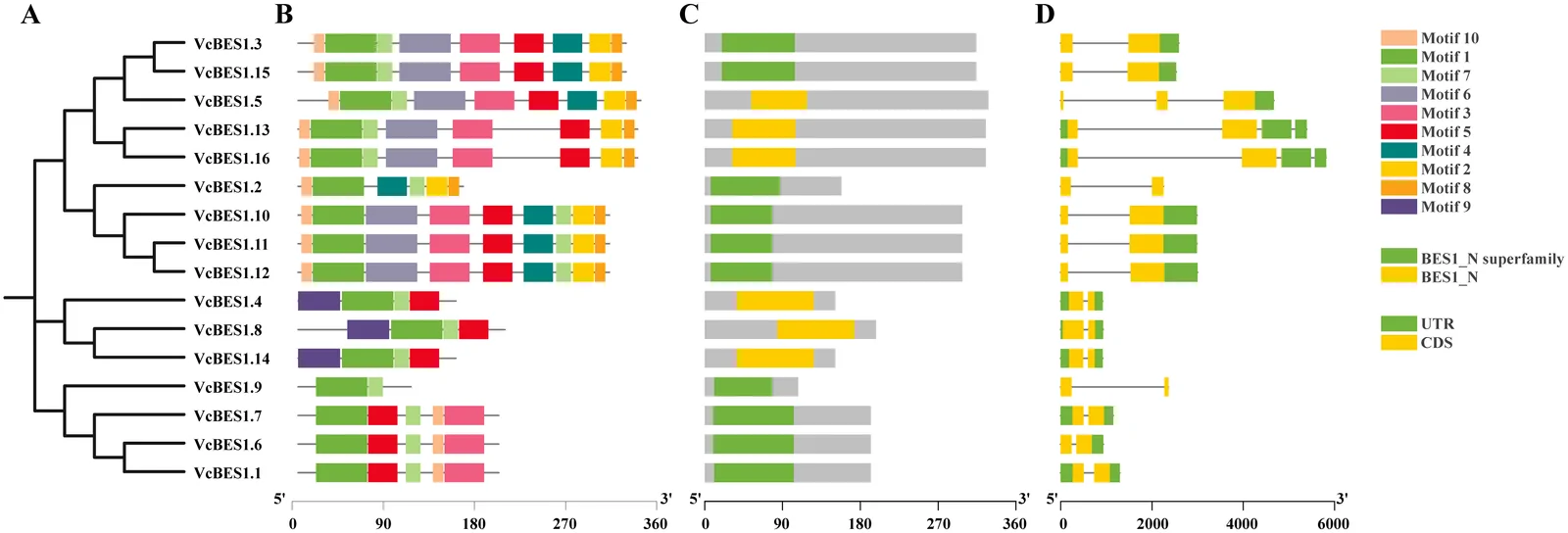
Characterization of *VcBES1* gene family in *Vaccinium corymbosum*. **(A)** Phylogenetic tree of VcBES1 proteins. **(B)** Conserved motif composition of VcBES1 proteins identified by MEME Suite. Ten motifs are represented by differently colored boxes. **(C)** Domain architecture of VcBES1 proteins. The BES1_N domain (PF05687) is indicated in green. **(D)** Exon-intron structure of the *VcBES1* transcription factors. Yellow boxes indicate exons. Green boxes indicate untranslated 5’ and 3’ regions. Grey lines indicate introns.

### 3.3 *Cis*-acting element analysis of VcBES1 promoters

The 2, 000-bp regions upstream of VcBES1 coding sequences (CDSs) were submitted to the PlantCARE database to predict the *cis*-acting elements (**Figure 3**). A total of 14 types of *cis*-elements were identified and grouped into stress-responsive, hormone-responsive, and development-related categories. Their distribution was not uniform across phylogenetic groups. Group 4 promoters contained more ARE (anaerobic induction) and ABRE (ABA-responsive) motifs than the other groups, both linked to abiotic stress signalling. Group 2 promoters harbored a higher proportion of development-associated elements. Across the family, ARE, ABRE, CGTCA-motif (MeJA-responsive), and MBS (drought-responsive MYB binding site) were the most abundant, suggesting transcriptional integration of hypoxia, ABA, jasmonate, and drought signals. The location information of these elements was listed in **Supplementary Table S2**.

**Figure 3:**
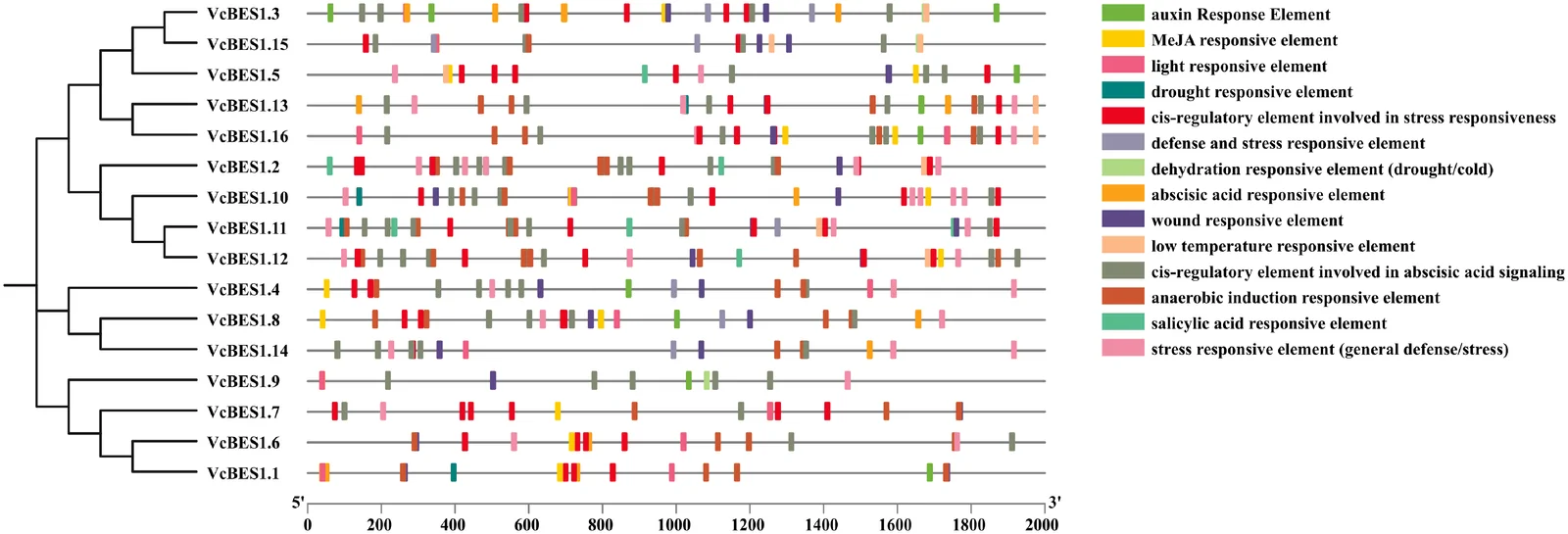
*Cis*-regulatory element analysis of *VcBES1* gene promoters. Different colored boxes represent different *cis*-acting elements.

### 3.4 Chromosomal distribution and gene duplication of the *VcBES1* family

We performed intragenomic collinearity analysis to trace the duplication evolutionary history of the *VcBES1* gene family. Fourteen collinear relationships consistent with segmental duplication were detected, involving 15 of the 16 *VcBES1* genes (**Figure 4**). The duplication network contained three fully connected triads (*VcBES1.3/1.5/1.15*; *VcBES1.4/1.8/1.14*; *VcBES1.1/1.6/1.7*), consistent with successive duplications from single ancestral loci. We also identified a four-gene cluster consisting of *VcBES1.2*, *VcBES1.10*, *VcBES1.11* and *VcBES1.12*. *VcBES1.10* and *VcBES1.11* reside on VaccDscaff30 and show high sequence similarity, but their relationship cannot be classified as tandem duplication given their genomic distance. *VcBES1.13* and *VcBES1.16* constituted an independent syntenic pair with no collinear links to other family genes, whereas *VcBES1.9* showed no significant collinear blocks against any other VcBES1 member. Most duplicated pairs evolved under purifying selection (Ka/Ks < 1), consistent with the evolutionary conservation of this transcription factor family (**Supplementary Table S3**).

**Figure 4:**
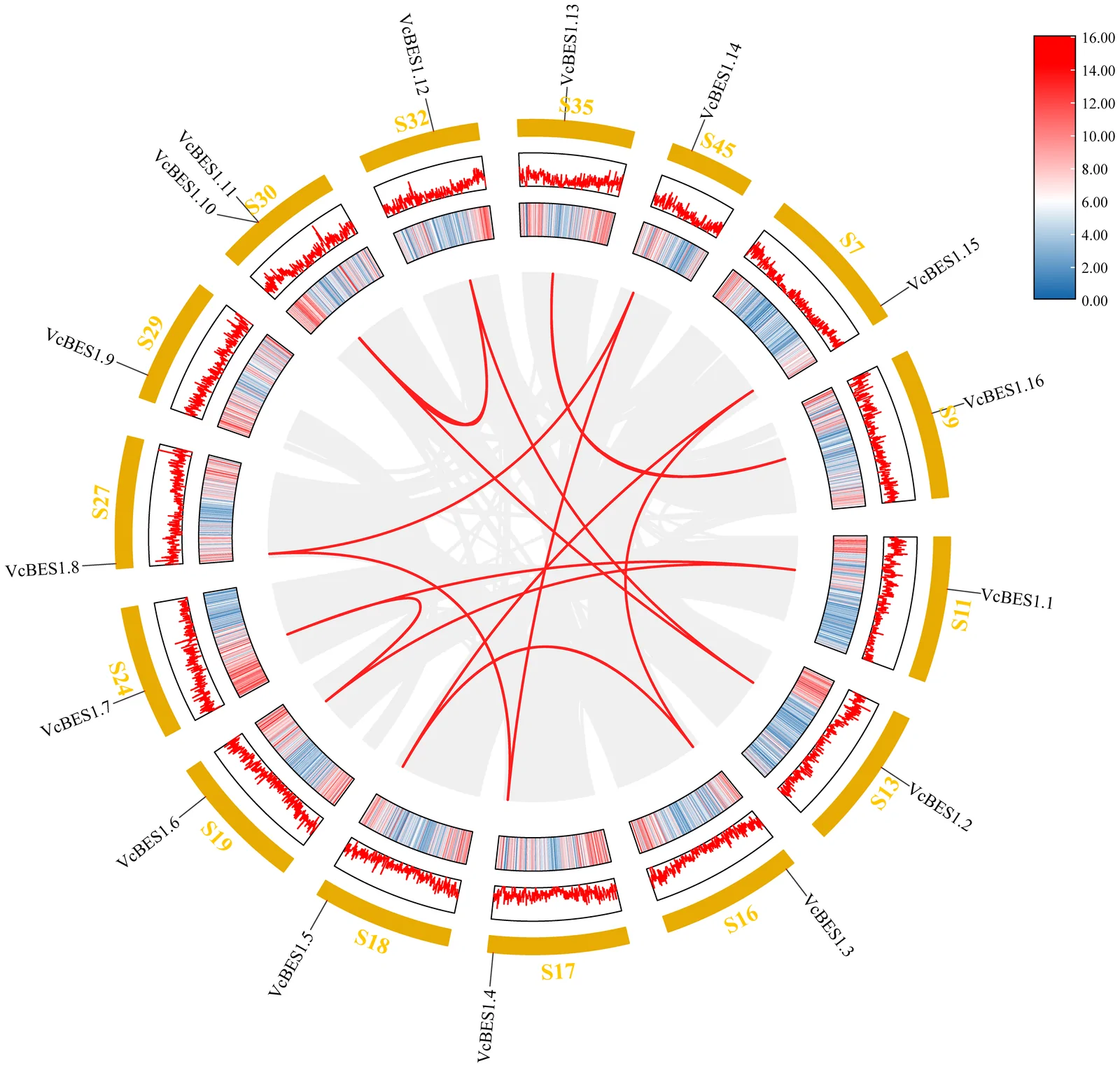
Intraspecific collinearity of the *VcBES1* gene family in blueberry. Yellow boxes denote scaffolds; red lines connect collinear *VcBES1* gene pairs. The heatmap along each scaffold shows gene density, with red indicating high density and blue indicating low density.

### 3.5 Synteny analysis of BES1 genes

We further performed synteny analyses of *V.corymbosum*, *A. thaliana*, *P. trichocarpa* and *S. lycopersicum* (**Figure 5**). A total of 23 collinear ortholog pairs were found between blueberry and poplar, 14 between blueberry and tomato, and only 11 between blueberry and *Arabidopsis* (**Supplementary Tables S4**–**S6**). Among these, six *VcBES1* genes retained collinear orthologues in all three comparator genomes. *VcBES1.3*, *VcBES1.5* and *VcBES1.15* correspond to *Arabidopsis* BR master regulators *AtBES1* and *AtBZR1*, while *VcBES1.2*, *VcBES1.11* and *VcBES1.12* cluster within the *BEH* subclade. Three genes (*VcBES1.4*, *VcBES1.8*, *VcBES1.14*) were conserved in tomato and poplar but absent from *Arabidopsis*, a pattern consistent with gene loss in the Brassicales lineage. The remaining seven genes, close to half the family, showed no interspecies synteny.

**Figure 5:**
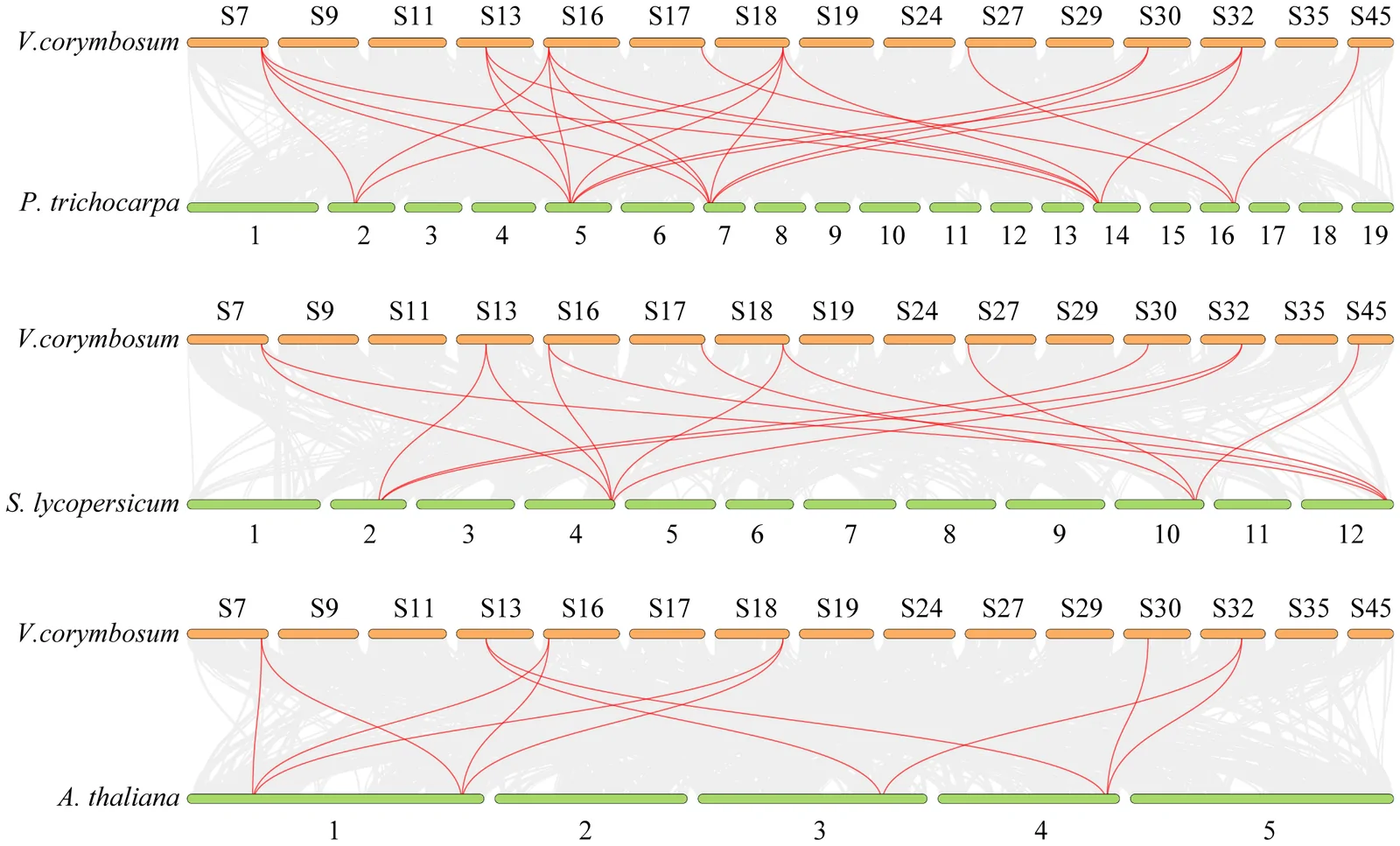
Cross-species collinearity of the BES1 gene family between blueberry (*Vaccinium corymbosum*) and three reference species: *Populus trichocarpa*, *Solanum lycopersicum*, and *Arabidopsis thaliana*. Orange strips denote the genomic scaffolds of *V. corymbosum*; green strips denote chromosomes of the reference species. Red lines connect syntenic BES1 orthologous pairs, delineating conserved syntenic blocks across the four genomes. The numbers on the chromosomes or scaffolds indicate chromosome or scaffold number.

### 3.6 Tissue-specific and stress-responsive expression patterns of the *VcBES1* family

RNA-seq transcriptome profiling was conducted to quantify tissue-specific expression and stress responses to drought and salinity. Tissue profiling revealed three expression modes (**Figure 6**). *VcBES1.10*, *VcBES1.11*, and *VcBES1.12* were constitutively abundant across all organs. *VcBES1.3*, *VcBES1.5*, and *VcBES1.15* showed root and stem preferential accumulation with low leaf expression. *VcBES1.4*, *VcBES1.8*, and *VcBES1.14* were essentially undetectable in all tissues examined.

**Figure 6:**
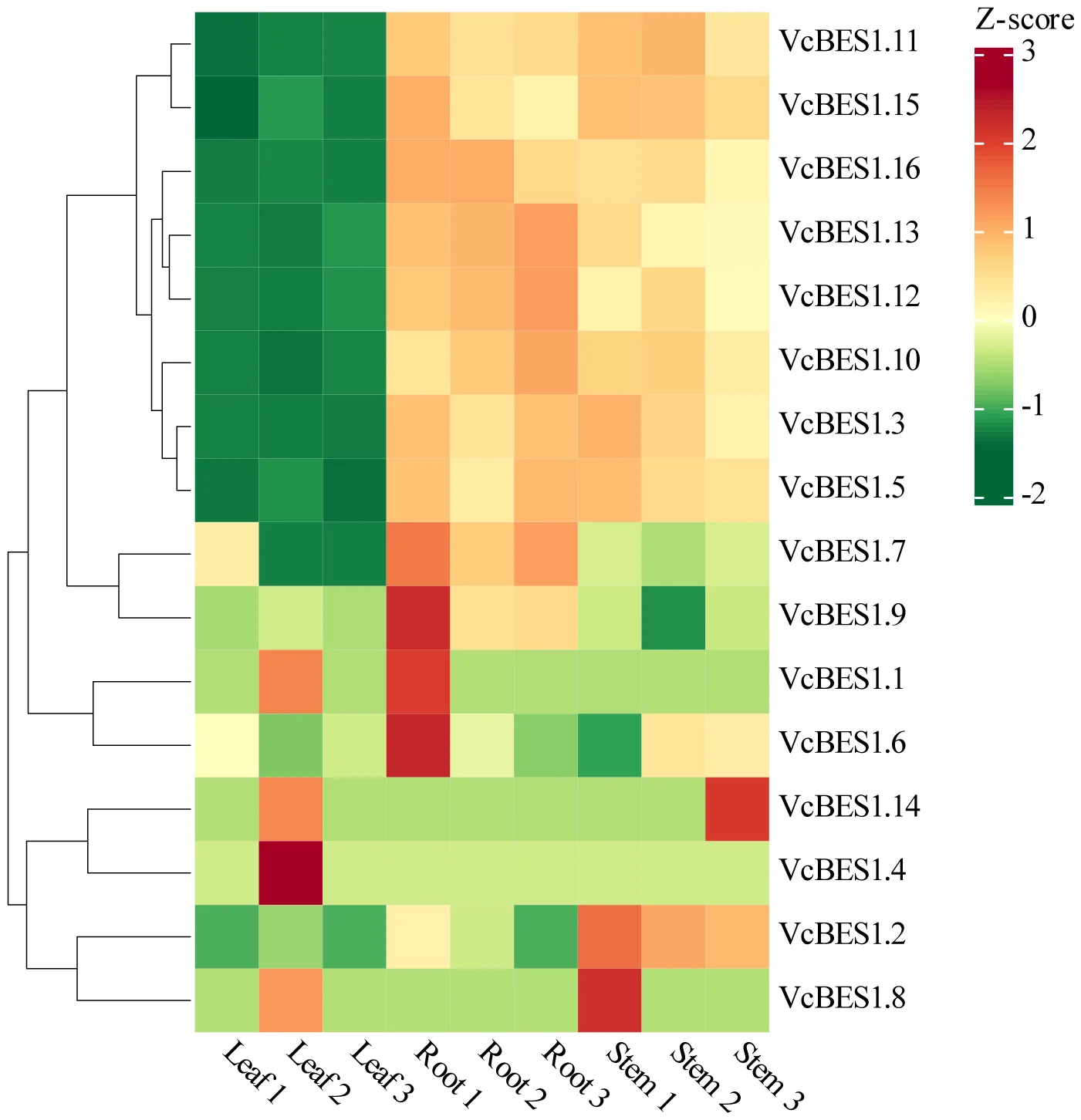
Tissue-specific expression patterns of *VcBES1s*. Transcript abundance (Z-score normalized TPM) is shown for leaf, root, and stem. The color gradient from green (low expression) to red (high expression) follows the scale bar on the right. Hierarchical clustering of genes is shown on the left.

We next characterized the expression patterns of *VcBES1s* under drought and salt treatments (**Figures 7**, **8**). Under drought stress, *VcBES1.2* and *VcBES1.3* were significantly up-regulated, while *VcBES1.1* and *VcBES1.7* were slightly down-regulated (**Figure 7**). Under salt stress, *VcBES1.3* and *VcBES1.4* were significantly up-regulated, while *VcBES1.8* and *VcBES1.9* were slightly down-regulated (**Figure 8**). Overall, most *VcBES1* members responded positively to the two abiotic stresses, highlighting the vital function of this gene family in blueberry drought and salt adaptation. Among these genes, *VcBES1.3* was the most strongly induced under both stresses, with log_2_FC = 3.95 under drought and a larger value of 4.93 under salt. Thus, *VcBES1.3* was selected for subsequent functional characterisation.

**Figure 7:**
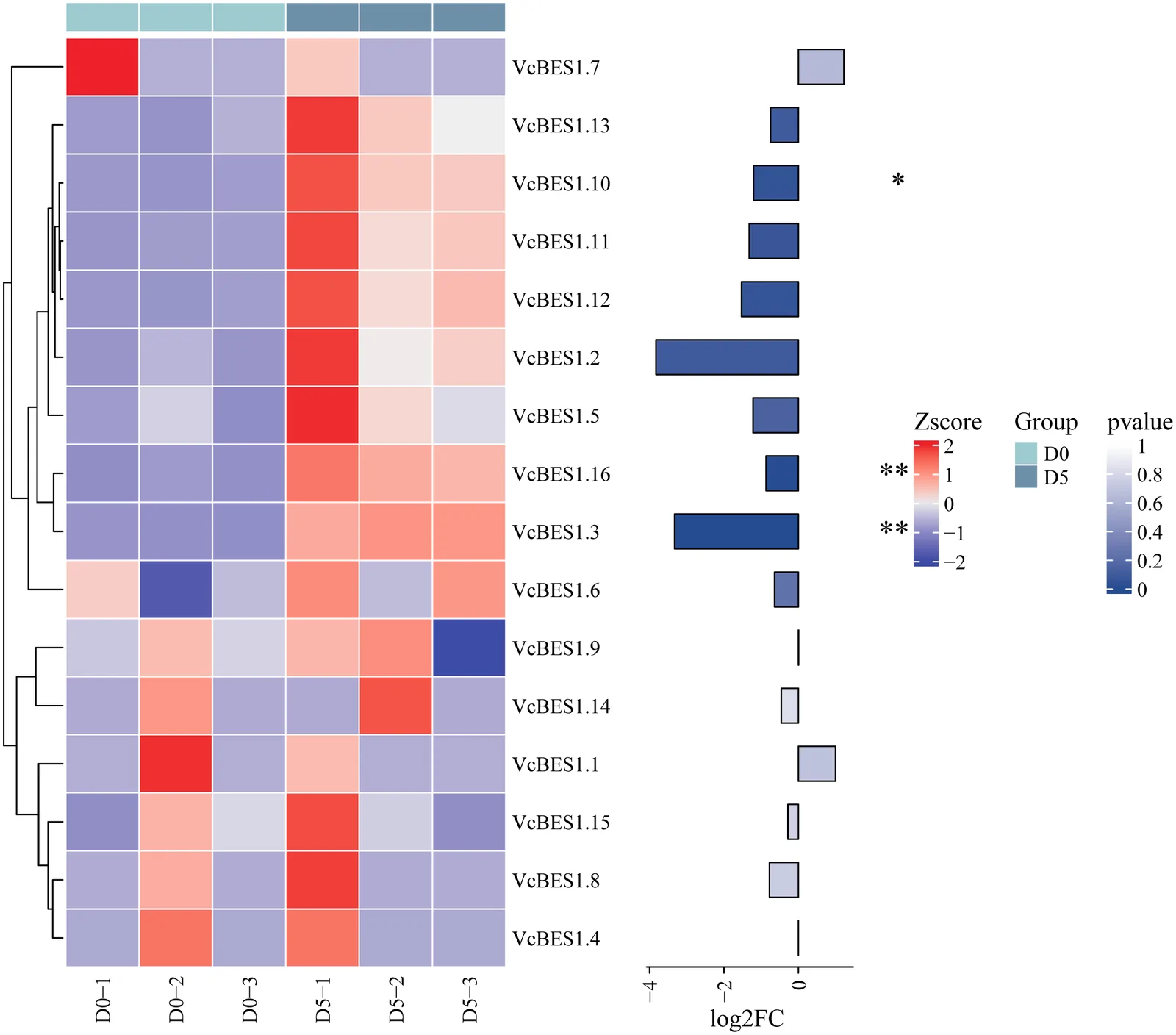
Differential expression profiling of *VcBES1* genes under drought stress treatment. The left heatmap shows transcript abundance (Z-score) of each *VcBES1* gene at day 0 (control) and day 5 (5 day water deprivation), with three biological replicates per group. Hierarchical clustering of genes is shown on the left. The right bar plot presents log_2_ fold changes between day 5 and day 0. Asterisks indicate significant differences (*p < 0.05, **p < 0.01). The color scale runs from blue (low expression) to red (high expression).

**Figure 8:**
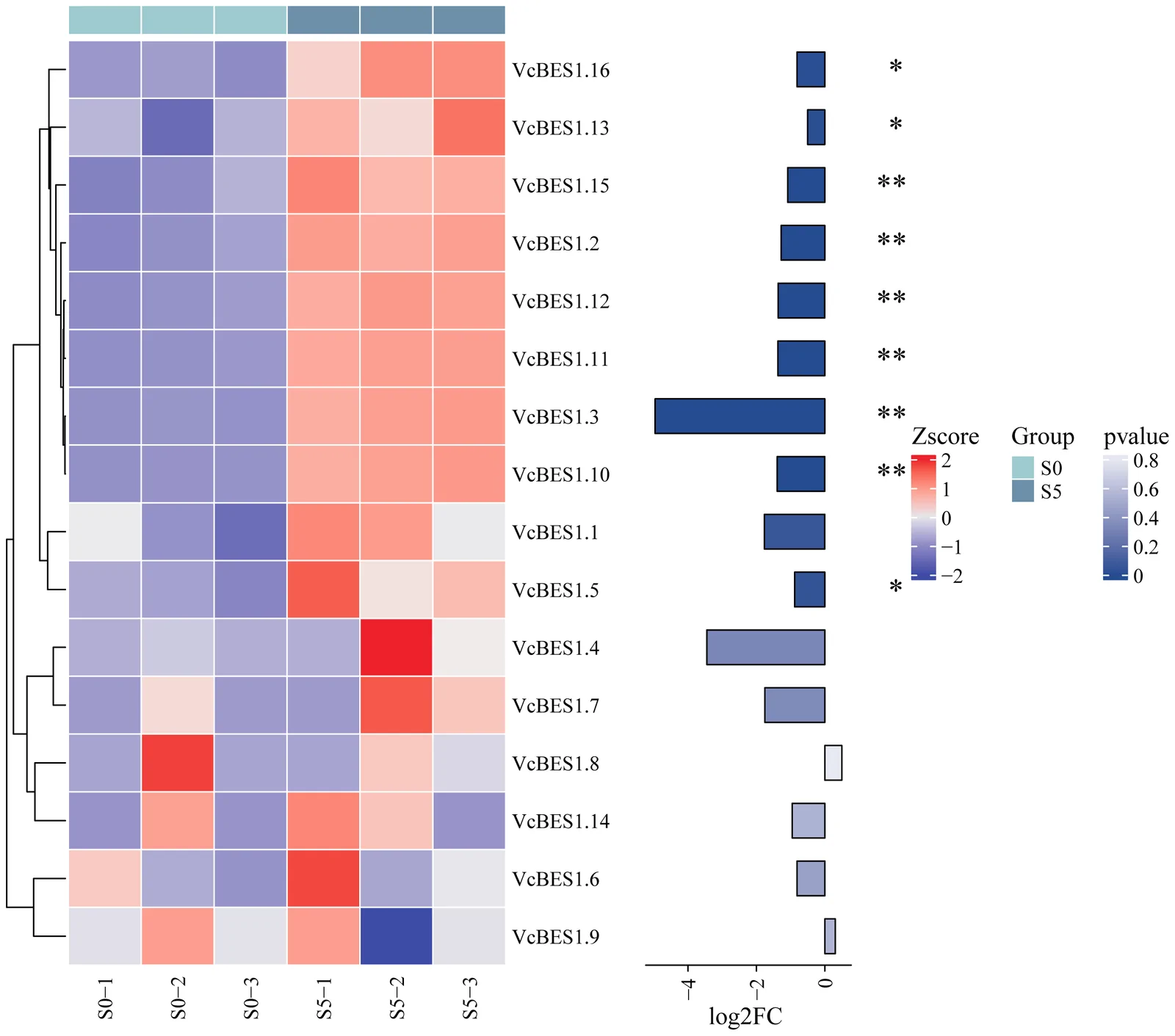
Differential expression profiling of *VcBES1* genes under salt stress treatment. The left heatmap shows transcript abundance (Z-score) of each *VcBES1* gene at day 0 (control) and day 5 (200 mM NaCl), with three biological replicates per group. Hierarchical clustering of genes is shown on the left. The right bar plot presents log_2_ fold changes between day 5 and day 0. Asterisks indicate significant differences (*p < 0.05, **p < 0.01). The color scale runs from blue (low expression) to red (high expression).

### 3.7 Subcellular localization of *VcBES1.3*

The *VcBES1.3* coding sequence was fused to EGFP and transiently expressed in tobacco leaf cells. Confocal microscopy showed full co-localization of the GFP signal with the nuclear marker H2B-mCherry, with no cytoplasmic fluorescence (**Figure 9A**). These observations indicated that VcBES1.3 proteins localized to the nucleus. Nuclear localisation is a prerequisite for transcription factor function.

**Figure 9:**
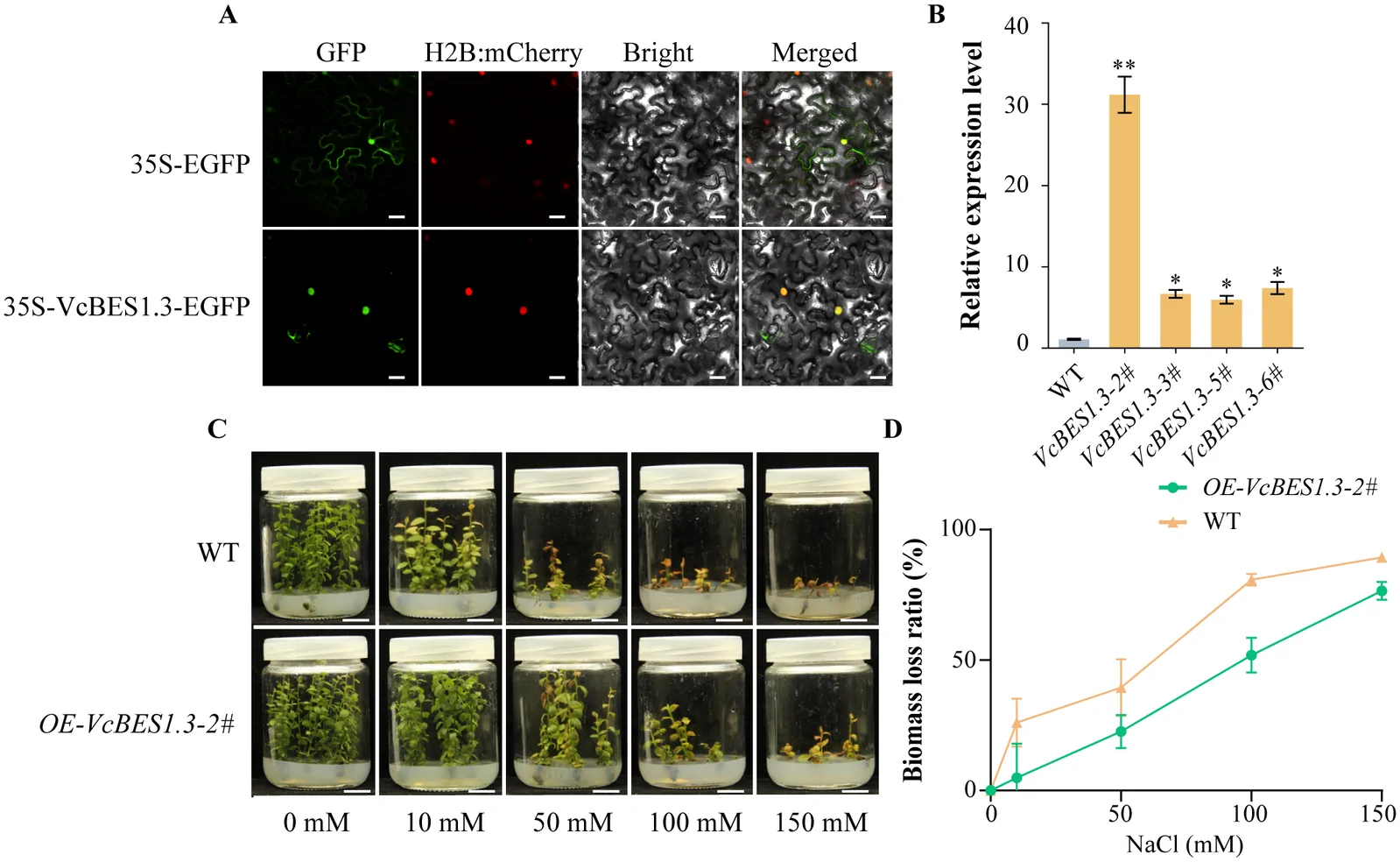
*VcBES1.3* is a nuclear protein that enhances salt tolerance in blueberry. **(A)** Subcellular localization of the VcBES1.3-EGFP fusion protein in *Nicotiana benthamiana* leaf epidermal cells. The empty vector was used as a control. Channels shown: GFP, H2B:mCherry (nuclear marker), bright field, and merged. Scale bars=20µm. **(B)** Transcript levels of *VcBES1.3* in wild-type (WT) and independent overexpression lines (*OE-VcBES1.3-2#, -3#, -5#, -6#*) measured by RT-qPCR. Data are mean ± SEM, n=3. Asterisks indicate significant differences relative to WT (Two-tailed Student’s *t*-test: *p < 0.05, **p < 0.01). **(C)** Phenotypes of WT and *OE-VcBES1.3-2#* plantlets grown on medium supplemented with 0, 10, 50, 100, or 150 mM NaCl for 60 days. **(D)** Biomass loss of WT and *OE-VcBES1.3-2#* plantlets under the NaCl gradients shown in **(C)**. The data are shown as mean  ± SEM; n =3 biological replicates, each comprising four plantlets (Two-way ANOVA, Tukey’s honestly significant difference (HSD) test, ***P < 0.001; **P < 0.01, ns, no significant difference).

### 3.8 Overexpression of *VcBES1.3* attenuates salt-induced growth inhibition in blueberry

To test the direct effect of *VcBES1.3* on plant’s salt response, we generated transgenic *V.corymbosum* overexpressing *VcBES1.3* for characterization. We obtained four independent *OE-VcBES1.3* lines. Among them, we selected the highest transgene overexpression line (*OE-VcBES1.3-2#*) for salt stress tests (**Figure 9B**). Following a 60-day gradient salt treatment (0, 10, 50, 100, and 150 mM), *OE-VcBES1.3-2#* exhibited superior growth performance across all tested salt concentrations (**Figure 9C**). At 100 mM NaCl, the transgenic line only experienced a 52.5% biomass loss, in contrast to an 81.0% biomass decline in WT (**Figure 9D**). At 150 mM NaCl, both genotypes were severely stunted (biomass loss exceeding 80%), although the OE line showed a slight, non-significant tendency toward lower biomass loss than the wild type. The *VcBES1.3*-mediated salt-protective effect was most pronounced at moderate stress intensity.

### 3.9 Silencing of *VcBES1.3* aggravates salt-induced oxidative damage in blueberry

To further investigate the function of *VcBES1.3* in salt tolerance, RNA interference (RNAi) was used to silence *VcBES1.3*. We obtained two independent RNAi lines, designated *RNAi-VcBES1.3-1#* and *RNAi-VcBES1.3-2#*. RT-qPCR analysis confirmed that *VcBES1.3* transcript levels were substantially reduced in both RNAi lines relative to WT (**Figure 10A**). After 12 days of treatment with 150 mM NaCl, *RNAi-VcBES1.3* plants developed severe leaf chlorosis and necrosis, whereas WT showed only mild salt spotting (**Figures 10B, C**).

**Figure 10:**
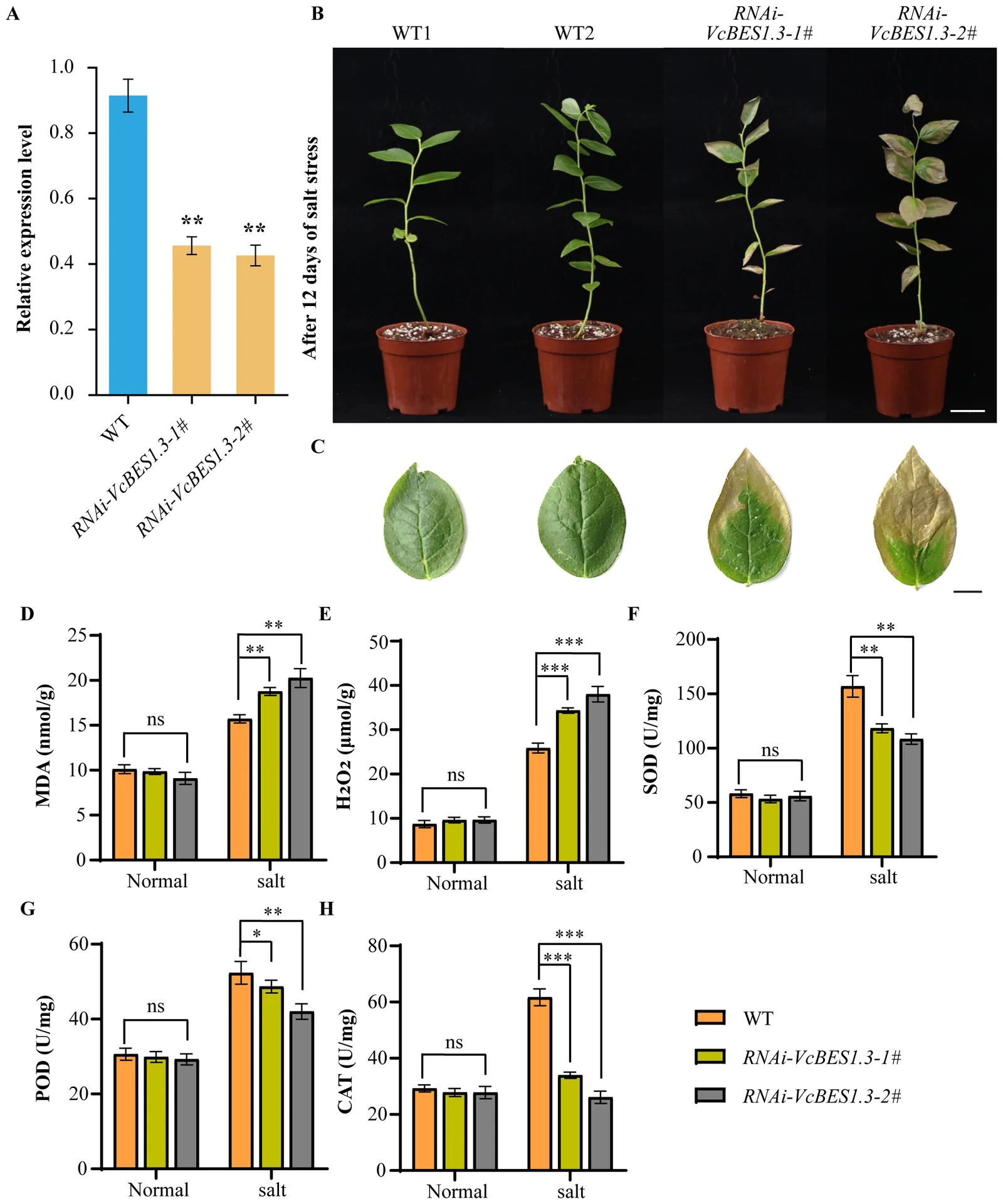
Silencing of *VcBES1.3* impairs salt tolerance and disrupts antioxidant metabolism in blueberry. **(A)** Transcript levels of *VcBES1.3* in wild-type (WT) and RNAi lines (*RNAi-VcBES1.3-1#, -2#*) measured by RT-qPCR. Data are mean ± SEM, n=3 biological replicates. Asterisks indicate significant differences relative to WT (Two-tailed Student’s *t*-test: **p < 0.01). **(B)** Whole-plant phenotypes of WT and *RNAi-VcBES1.3* lines after 12 days of salt stress (150mM NaCl). Three independent biological replicates were performed, with at least six plants per genotype per replicate. Scale bars=5 cm. **(C)** Detached leaves from the plants shown in **(B)**, illustrating salt-induced foliar damage in each genotype. Scale bars=2 cm. **(D–H)** Physiological measurements in WT and RNAi lines under normal and salt stress conditions. **(D)** MDA content. **(E)** H_2_O_2_ content. **(F)** SOD activity. **(G)** POD activity. **(H)** CAT activity. For all panels, bars are colored as follows: WT (orange), *RNAi-VcBES1.3-1#* (yellow), *RNAi-VcBES1.3-2#* (gray). The data are shown as mean ± SEM.; n=3 biological replicates (One-way ANOVA followed by Tukey’s HSD test within each treatment; *P < 0.05; **P < 0.01; ***P < 0.001; ns, no significant difference).

Physiological measurements including POD, SOD, CAT, MDA, and H_2_O_2_ revealed that RNAi plants were significantly impaired under salt stress compared to WT (**Figures 10D–H**). Under normal conditions, none of the measured parameters differed between *RNAi-VcBES1.3* lines and WT. Upon NaCl treatment, MDA and H_2_O_2_ contents were markedly elevated in silenced plants. In contrast, the activities of POD, SOD and CAT were significantly lower in RNAi lines under salt stress. These results demonstrate that silencing of *VcBES1.3* aggravates salt damage in blueberry. Combined with the enhanced salt tolerance observed in *VcBES1.3* overexpressing plants, these results confirm that *VcBES1.3* positively regulates blueberry salt tolerance.

## 4 Discussion

The BES1 family is known from model plants and several crops (Yin et al., 2005; Bai et al., 2007; Song et al., 2018; Yu et al., 2018; Su et al., 2021). We identified 16 *VcBES1* genes, a number that sits between the family sizes reported for tomato (9) and apple (22). All four groups previously defined in *Arabidopsis*, rice, and poplar are represented in blueberry (Cao et al., 2020; Su et al., 2021). The four groups therefore predate the rosid-asterid split. This is consistent with the early land-plant origin of the BES1 family inferred from phylogenomic analyses (Sun et al., 2022).

Interspecies collinearity resolved the family into three groups. Six *VcBES1* genes are conserved across tomato, *Arabidopsis*, and poplar and define what can be considered the ancestral eudicot core; three of these reside in genomic blocks syntenic with AtBES1 and AtBZR1 (**Figure 5**). Another three genes appeared in tomato and poplar but were missing from *Arabidopsis*. Poplar and *Arabidopsis* shared a common ancestor before the Salicoid whole-genome duplication (Tuskan et al., 2006), so the absence of these genes from *Arabidopsis* is best explained by gene loss in the Brassicales lineage. The remaining seven *VcBES1* genes lacked interspecies collinearity. At 43.8% of the family, this lineage-restricted fraction exceeds the conserved core. Gene duplications drive transcription factor family expansion (Cannon et al., 2004; Freeling, 2009), and the observed pattern is consistent with amplification within the Ericales after divergence from the rosid lineage. High-quality chromosome-scale assemblies for closely related *Vaccinium* species remain scarce, which limits broader phylogenetic comparisons at this stage. The proportion of lineage-restricted genes suggests that the BES1 family in this species may have diversified through lineage-specific processes that warrant further investigation.

Based on our RNA-seq data, *VcBES1.3* was the most strongly stress-responsive gene in the BES1 family, showing log_2_ fold changes between 3.95 and 4.93 under drought and salt treatments (**Figures 7**, **8**). By contrast, other *VcBES1* members showed considerably weaker responses or no detectable changes. *Overexpression* of *VcBES1.3* improved salt tolerance, with transgenic plants retaining more biomass under salt stress (**Figure 9**). Silencing of *VcBES1.3* reduced salt tolerance, resulting in an earlier collapse of the antioxidant system under saline conditions (**Figure 10**). *BES1* directly regulates ion transporters, including *NHX2* and *CAX3*, under salt stress (Feng et al., 2023a), and *BES1/BZR1* coordinate growth and stress responses through *WRKY*, *RD26*, and other transcription factors (Cao et al., 2024; Li et al., 2025). Manipulation of this pathway at the level of *GSK1* improved biomass and grain yield in barley under salt stress (Kloc et al., 2024), and sustained expression of *VcBES1.3* may play a comparable role in blueberry by allowing plants to maintain growth under salt stress. However, the molecular targets of *VcBES1.3* in blueberry remain unknown, and the present data do not distinguish between direct and indirect effects on the antioxidant system or ion homeostasis.

Gradient salt treatment revealed a dose-dependent effect. The OE line retained more biomass than the wild type at all NaCl concentrations tested, with the largest difference at 100 mM: biomass loss was 52.5% in the transgenic line versus 81.0% in the wild type (**Figures 9C, D**). At 150 mM NaCl, both genotypes were severely stunted, and the advantage of the OE line narrowed. *VcBES1.3*-mediated protection is therefore most effective at moderate stress intensity and tapers off under extreme saline conditions. In the RNAi lines, antioxidant enzyme induction was suppressed. Catalase was among the most affected activities. H_2_O_2_ and lipid peroxidation products accumulated (**Figures 10D–H**). These observations point to oxidative damage as one factor underlying the salt sensitivity of the silenced plants, though whether *VcBES1.3* controls antioxidant genes directly or through intermediate factors is not known. The phenotype aligns with the coordinating role of *BES1/BZR1* in stress-responsive gene networks (Cao et al., 2024; Li et al., 2025).

The functional evidence for *VcBES1.3* rests on a single gene. *VcBES1.10*, *VcBES1.11*, and *VcBES1.12*, all constitutively expressed Group 4 members, may also participate in salt tolerance and were not examined here. The mechanistic basis of *VcBES1.3* function has not been resolved: whether it acts directly on the antioxidant system or on ion homeostasis (e.g., Na^+^/K^+^ balance and the expression of NHX, CAX, and SOS pathway genes) remains unknown Its direct transcriptional targets and interacting partners remain unidentified and will require chromatin immunoprecipitation and protein interaction assays to define. Our functional assays focused on salt stress. While transcriptomic data indicate that *VcBES1.3* exhibits a similar response under drought (**Figure 8**), corresponding functional verification remains to be performed in future work.

We report the first characterisation of the *VcBES1* transcription factor family in blueberry. *VcBES1.3* is a positive regulator of salt tolerance. The gain- and loss-of-function evidence points to *VcBES1.3* as a candidate for molecular breeding of stress-resilient blueberry cultivars.

## Data Availability

The datasets presented in this study can be found in online repositories. The raw RNA-seq data have been deposited in the Genome Sequence Archive (GSA) of the National Genomics Data Center, China National Center for Bioinformation, under accession numbers CRA049980 and CRA049981 (https://ngdc.cncb.ac.cn/gsa).The datasets presented in this study can be found in thearticle/**Supplementary Material**.
