# Supplementary material for: Genome-wide identification of the BES1 gene family and functional study of VcBES1.3 under salt stress in blueberry (Vaccinium corymbosum)
Source: Front Plant Sci. 2026 Oct 13;17:1965422. doi: 10.3389/fpls.2026.1965422 (PMC13709998; doi:10.3389/fpls.2026.1965422)
Supplement: Supplementary file 1 [file Supplementaryfile2.docx]

Supplementary Material

## Supplementary Figures

**Supplementary Figure 1. Salt-tolerance analysis of the additional overexpression line *OE-VcBES1.3-6#*.** (A) Phenotypes of WT and *OE-VcBES1.3-6#* plantlets grown on medium supplemented with 0, 10, 50, 100, or 150 mM NaCl for 60 days. (B) Biomass loss of WT and *OE-VcBES1.3-6#* plantlets under the NaCl gradients shown in (A). The data are shown as mean ±SEM; n =3 biological replicates, each comprising four plantlets (Two-way ANOVA, Tukey’s honestly significant difference (HSD) test, ****P* < 0.001; ***P* < 0.01, ns, no significant difference).

**Supplementary Figure 2. Expression of the 16 *VcBES1* genes in the two RNAi lines and the wild type.** Relative expression of all 16 *VcBES1* genes in the wild type (WT), *RNAi-1#*, and *RNAi-2#* lines determined by qRT-PCR. Data are means ± SEM (n = 3 biological replicates). One-way ANOVA followed by Tukey's HSD test; ****P* < 0.001; ns, no significant difference.
